# Effectiveness of direct-acting antiviral therapy for hepatitis C in difficult-to-treat patients in a safety-net health system: a retrospective cohort study

**DOI:** 10.1186/s12916-017-0969-3

**Published:** 2017-11-20

**Authors:** Christina Yek, Carolina de la Flor, John Marshall, Cindy Zoellner, Grace Thompson, Lisa Quirk, Christian Mayorga, Barbara J. Turner, Amit G. Singal, Mamta K. Jain

**Affiliations:** 1 Parkland Health and Hospital System, Dallas, Texas USA; 2 University of Texas Southwestern Medical Center, Dallas, Texas USA; 3 University of Texas Health Science Center, San Antonio, Texas USA

**Keywords:** Chronic hepatitis C, Direct-acting antiviral therapy, Real-world cohort, Safety-net hospital, Indigent population

## Abstract

**Background:**

Direct-acting antivirals (DAAs) have revolutionized chronic hepatitis C (HCV) treatment, but real-world effectiveness among vulnerable populations, including uninsured patients, is lacking. This study was conducted to characterize the effectiveness of DAAs in a socioeconomically disadvantaged and underinsured patient cohort.

**Methods:**

This retrospective observational study included all patients undergoing HCV treatment with DAA-based therapy between April 2014 and June 2016 at a large urban safety-net health system (Parkland Health and Hospital System, Dallas, TX, USA). The primary outcome was sustained virologic response (SVR), with secondary outcomes including treatment discontinuation, treatment relapse, and loss to follow-up.

**Results:**

DAA-based therapy was initiated in 512 patients. The cohort was socioeconomically disadvantaged (56% uninsured and 13% Medicaid), with high historic rates of alcohol (41%) and substance (50%) use, and mental health disorders (38%). SVR was achieved in 90% of patients (n = 459); 26 patients (5%) were lost to follow-up. SVR was significantly lower in patients with decompensated cirrhosis (82% SVR; OR 0.37, 95% CI 0.16–0.85) but did not differ by insurance status (*P* = 0.98) or alcohol/substance use (*P* = 0.34). Reasons for treatment failure included loss to follow-up (*n* = 26, 5%), viral relapse (*n* = 16, 3%), non-treatment-related death (*n* = 7, 1%), and treatment discontinuation (*n* = 4, 1%). Of patients with viral relapse, 6 reported non-compliance and have not been retreated, 5 have been retreated and achieved SVR, 4 have undergone resistance testing but not yet initiated retreatment, and 1 was lost to follow-up.

**Conclusions:**

Effective outcomes with DAA-based therapy can be achieved in difficult-to-treat underinsured populations followed in resource-constrained safety-net health systems.

**Electronic supplementary material:**

The online version of this article (doi:10.1186/s12916-017-0969-3) contains supplementary material, which is available to authorized users.

## Background

Chronic hepatitis C virus (HCV) infects an estimated 3.4–6.0 million people in the United States and approximately 71 million people worldwide [1, 2]. Deaths related to HCV infection have been on the rise for the past decade [3], with cirrhosis-related complications, including hepatocellular carcinoma (HCC), accounting for the increased morbidity and mortality [4, 5]. However, a sustained virologic response (SVR) is associated with improved quality of life as well as reduced rates of fibrosis progression, cirrhosis-related complications, HCC, and all-cause mortality [5, 6].

Prior to the advent of direct-acting antiviral (DAA) therapy, the mainstay of HCV therapy involved interferon (IFN)-based regimens that had frequent contraindications, were poorly tolerated, and achieved at best a 50% SVR rate [7]. Introduction of DAAs has since revolutionized the HCV treatment landscape. SVR rates for DAA therapy exceed 90% in registration trials and are better tolerated than IFN-based regimens [8]. Similarly, real-world data suggests high rates of effectiveness in clinical practice; however, studies thus far have described outcomes primarily in academic centers treating well-insured, primarily Caucasian populations [9–12].

A large burden of HCV infection falls on marginalized populations, including the uninsured, homeless, incarcerated, substance users, those with mental disorders, and those infected with HIV [1, 13]. However, there is a paucity of data regarding effectiveness of DAAs in these populations, who are largely treated in safety-net health systems. Further, DAAs may be unavailable for these patients due a myriad of barriers, including high drug costs, limited healthcare access, low health literacy, language barriers, unstable housing, and significant psychosocial and medical comorbidities [14]. Additionally, at-risk populations have been reported to have lower rates of follow-up, which may translate into undetected drug-related adverse events [15], poor adherence that could lead to treatment failure, and risky behavior such as ongoing substance abuse that may be associated with an increased likelihood of reinfection [16]. Consequently, these patients are often excluded from registration trials [17]. Thus, real-world studies in these vulnerable populations are necessary to inform interventions that address the specific challenges faced in their care.

The aim of our study is to describe the effectiveness of DAA-based HCV therapy in a racially diverse, socioeconomically disadvantaged, underinsured population seen in a large urban safety-net health system.

## Methods

### Study setting and population

This is a retrospective study of patients starting IFN-free, DAA-based treatment for chronic HCV in the Parkland Health and Hospital System (PHHS), the safety-net health system of Dallas, TX, USA. PHHS is an integrated system with 13 primary care provider clinics in low-income neighborhoods, a hepatology outpatient clinic, and a tertiary-care hospital. Although PHHS cares for a high proportion of uninsured and underinsured individuals, these patients are able to access medical care, including HCV- and liver-related treatment, through a county-funded subsidy program.

We included all HCV-infected patients initiating DAA-based, IFN-free therapy between April 2014 and June 2016. The study period started after April 2014, as this post-dated CDC and USPSTF recommendations for HCV screening and availability of DAA therapy for all genotypes. During the study period, 2078 patients with chronic HCV were seen in our hepatology clinic, of whom the majority (76%) was aged 46–65 years and over two-thirds were racial/ethnic minorities (45% Black, 21% Hispanic, 29% White); 37% of these patients were uninsured, while 30% had Medicaid, 28% Medicare, and 5% commercial insurance. Patients were defined as having a history of mental health disorder if they had chart documentation of ICD-10 codes for schizophrenia, schizotypal and delusional disorders (F20–F29), mood (affective) disorders (F30–F39), or neurotic, stress-related, and somatoform disorders (F40–F48). Substance use was determined based on chart documentation of recreational drug use or of mental and behavioral disorders due to substance abuse (ICD-10 codes F11–F16, F18, F19). Alcohol abuse was defined as consumption of more than 7 drink-equivalents per week for women and more than 14 drink-equivalents per week for men, or an ICD-10 diagnosis of alcohol-related disorder (F10). The institutional review board of UT Southwestern approved this study.

### Hepatitis C clinic structure and staff

**Fig. 1:**
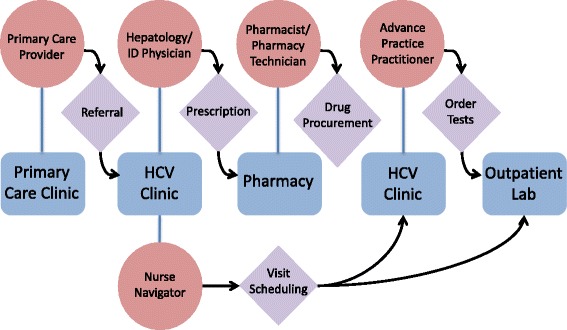
Schematic model of patient flow. Flow chart depicting patient movement through the HCV clinic from time of referral to assessment of treatment response at the end of therapy. *ID* infectious diseases, *HCV* hepatitis C virus, *Lab* laboratory (phlebotomy)

All patients underwent HCV treatment evaluation and follow-up in the PHHS outpatient hepatology clinic. Clinic staff consisted of 0.4 full-time equivalents (FTE) of infectious diseases or hepatology faculty physicians, 0.2 FTE advanced practice practitioners, 0.5 FTE nurse navigators, and 0.2 FTE pharmacist/pharmacy technicians with clinics held once weekly. Resources were subsequently increased to include 0.3 FTE advanced practice practitioners, 1.0 FTE nurse navigators, and 0.4 FTE pharmacist/pharmacy technicians with clinics held four times a week starting in February 2016 (Fig. 1).

Treatment regimens were selected by providers following evidence-based guidelines [18] that consider HCV genotype, presence of cirrhosis, previous HCV treatment failure, presence of chronic kidney disease, and insurance formulary restrictions. Treatment regimens during the study period included (1) sofosbuvir (SOF) and ribavirin (RBV), (2) SOF and ledipasvir (LDV) ± RBV, (3) SOF and simeprevir (SMV) ± RBV, (4) SOF and daclatasvir (DCV) ± RBV, (5) ombitasvir (OBV), ritonavir-boosted paritaprevir (PTV/r), and dasabuvir (DSV) ± RBV, or (6) elbasvir (EBR) and grazoprevir (GZR).

Pharmacists or pharmacy technicians completed prior authorization paperwork for insured patients and applications for patient assistance programs (PAPs) through pharmaceutical companies for uninsured patients. The nurse navigator scheduled follow-up appointments and laboratory testing. Nurse practitioners saw most patients on treatment, assessing for compliance, on-treatment response, and any adverse effects. Infectious disease or hepatology faculty followed patients with organ transplantation, HIV co-infection, or decompensated cirrhosis.

### HCV treatment evaluation and monitoring

Patients completed baseline laboratory testing including complete blood count, renal and hepatic function panels, coagulation tests, HCV genotype, and HCV viral load. Cirrhosis was defined as stage 4 liver fibrosis determined by non-invasive markers of fibrosis (FibroSure or FibroTest) [19, 20], liver biopsy, or imaging (ultrasound, computed tomography or magnetic resonance imaging) showing a cirrhotic-appearing liver combined with clinical signs of portal hypertension. Patients with cirrhosis had hepatic imaging prior to treatment, typically with ultrasound, to evaluate for presence of HCC. Decompensated cirrhosis was defined as history of ascites, hepatic encephalopathy, bleeding esophageal varices, or HCC. Patients had laboratory testing, including complete blood count, renal and hepatic function panels, and HCV viral load, every 4 weeks during treatment and 12 weeks after treatment completion to assess for SVR.

### Statistical analysis

The primary outcome of this study was SVR, defined as undetectable plasma HCV RNA at least 12 weeks after the end of HCV treatment. Secondary outcomes included premature discontinuation of HCV treatment, relapse after HCV treatment, and loss to follow-up prior to SVR assessment. Viral relapse was defined as undetectable viral load at the end of DAA treatment but subsequent detectable viral load at 12 weeks after treatment end. We compared outcomes across a priori defined subgroups based on race/ethnicity, insurance status, fibrosis stage, and HCV treatment regimen. Comparisons of categorical values between groups were performed using the χ^2^ test with Yates correction. Predictors of SVR were identified using univariate and multivariate logistic regression analyses. Statistical significance was defined as *P* < 0.10 for univariate and *P* < 0.05 for multivariate analyses. Statistical analysis was conducted using Stata 11.0 (College Station, TX, USA).

## Results

### Study population

Between April 2014 to June 2016, DAA-based therapy was initiated in 512 patients. Baseline characteristics of the patients receiving treatment showed a median age of 58 years, with the majority (56%) being male (Table 1). Our cohort was racially diverse, with 44% Black, 36% White, and 16% Hispanic patients, and represented a socioeconomically disadvantaged population with 56% of patients being uninsured and 13% covered by Medicaid insurance. Over one-third of patients had a history of mental health disorders, and two-thirds reported a history of alcohol or drug use, with 141 (28%) reporting both alcohol and substance use histories. Approximately half of patients (*n* = 262, 51%) had cirrhosis, of which 21% (*n* = 56) had hepatic decompensation. Most patients were treatment naïve, with only 16% (*n* = 80) having failed previous treatment with IFN-based therapy. Co-infection with HIV and HBV were present in 11% (*n* = 54) and 3% (*n* = 15), respectively. All HIV-infected patients were receiving antiretroviral therapy; antiretroviral regimens were changed in 16 patients to avoid interaction with DAA therapy. The most common HCV genotypes were genotype 1a (*n* = 309, 60%) and genotype 1b (*n* = 99, 19%). The most common treatment regimens were LDV + SOF ± RBV (*n* = 355, 69%), followed by SOF + RBV (n = 52, 10%), and SMV + SOF ± RBV (*n* = 48, 9%) (Additional file 1: Table S1).

**Table 1:** Baseline characteristics of all patients; comparison of insured and uninsured patients

Baseline characteristics	Patients, N	SVR, N	Uninsured, N	Insured, N	*P* ^a^
	(% of all patients)	(% of group)	(% of uninsured)	(% of insured)	
All patients	512	459 (90)	289 (56)	223 (44)	NS
Sex, Male	288 (56)	252 (88)	158 (55)	130 (58)	NS
Race					**
White	185 (36)	161 (87)	114 (39)	71 (32)	
Black	223 (44)	200 (90)	107 (37)	116 (52)	
Hispanic	81 (16)	76 (94)	50 (17)	31 (14)	
Other	23 (5)	22 (96)	18 (6)	5 (2)	
Age, Median (interquartile range)	58 (54–62)	NA	57 (53–61)	59 (54–64)	**
BMI, Median (interquartile range)	28 (25–33)	NA	28 (25–33)	28 (25–34)	NS
Treatment-experienced	80 (16)	75 (94)	36 (12)	44 (20)	*
Cirrhosis	262 (51)	227 (87)	133 (46)	129 (58)	*
Decompensated	56 (11)	46 (82)	27 (9)	29 (13)	NS
HCV genotype					NS
Genotype 1a	309 (60)	273 (88)	168 (58)	141 (63)	
Genotype 1b	99 (19)	91 (92)	54 (19)	45 (20)	
Genotype 2	45 (9)	41 (91)	25 (9)	18 (8)	
Genotype 3	30 (6)	27 (90)	23 (8)	7 (3)	
Other genotypes	29 (6)	29 (100)	19 (7)	10 (4)	
Treatment regimen					NS
SOF + RBV	52 (10)	46 (88)	30 (10)	22 (10)	
SMV + SOF ± RBV	48 (9)	39 (81)	21 (7)	27 (12)	
DCV + SOF ± RBV	26 (5)	23 (88)	15 (5)	11 (5)	
LDV + SOF ± RBV	355 (69)	323 (91)	214 (74)	141 (63)	
OBV + PTV/r + DSV ± RBV	27 (5)	24 (89)	7 (2)	20 (9)	
EBR + GZR	4 (1)	4 (100)	2 (1)	2 (1)	
HIV co-infected	54 (11)	47 (87)	21 (7)	33 (15)	**
Chronic HBV	15 (3)	14 (93)	7 (2)	8 (4)	NS
Liver or kidney transplant	23 (4)	20 (87)	9 (3)	14 (6)	NS
History of alcohol abuse	208 (41)	183 (88)	109 (38)	99 (44)	NS
History of drug abuse	255 (50)	226 (89)	141 (49)	114 (51)	NS
History of mental health disorder	194 (38)	172 (89)	113 (39)	81 (36)	NS

^a^ *P* value of uninsured versus insured by χ^2^ test for categorical variables and Mann–Whitney test for continuous variables; *P < 0.05; **P < 0.01

*BMI* body mass index, *HCV* hepatitis C virus, *Other genotypes* genotypes 4 & 6 or patients infected with one or more genotypes, *SOF* sofosbuvir, *RBV* ribavirin, *SMV* simeprevir, *DCV* daclatasvir, *LDV* ledipasvir, *OBV* ombitasvir, *PTV/r* ritonavir-boosted paritaprevir, *DSV* dasabuvir, *EBR* elbasivr, *GZR* grazoprevir, *HIV* human immunodeficiency virus, *HBV* hepatitis B virus infection, *SVR* sustained virologic response, *NA* not available, *NS* not significant

There were notable differences between insured and uninsured patients (Table 1). Insured patients were significantly older (median age 59 vs. 57 years, *P* < 0.001), more likely of Black race (52% vs. 37%, *P* < 0.001), cirrhotic (58% vs. 46%, *P* = 0.008), treatment-experienced (20% vs. 12%, *P* = 0.03), and HIV co-infected (15% vs. 7%, *P* = 0.009).

### Treatment outcomes

Of 512 total patients, SVR occurred in 459 patients (SVR rate of 90% by intention-to-treat analysis, 94% per protocol analysis). Twenty-six patients (5%) were lost to follow-up. Five of the 26 patients lost to follow-up subsequently visited their primary care provider but failed to receive repeat viral load testing. Of these patients, 3 had documented biochemical response with normalized liver function tests but no viral load to confirm SVR, and 1 had negative viral load 8 weeks after treatment but no documentation of SVR.

Twenty-seven patients had treatment failure due to premature discontinuation or relapse. Seven (1.4%) patients died during treatment, including 4 from cirrhosis-related complications (hepatorenal syndrome, portopulmonary hypertension, cholangiocarcinoma, and variceal bleed) and 3 from non-liver-related diseases (pulmonary embolism, suicide, and unknown cause). Treatment was discontinued prematurely due to patient-reported adverse effects in 4 (0.8%) patients, including angioedema (n = 1), severe anemia (n = 1), and severe nausea/vomiting (n = 2). Sixteen (3.1%) patients completed HCV therapy but relapsed at the time of SVR assessment.

Of the 16 patients with HCV treatment relapse, 7 (44%) were treated with older regimens that are no longer standard-of-care, including SIM + SOF (n = 4) and SOF + RBV (n = 3). Six (38%) patients reported non-compliance with antiviral therapy and were not retreated. Five of the remaining 10 patients underwent testing for viral resistance mutations; 4 patients with HCV genotype 1 treated with LDV + SOF had NS5A resistance mutations and are yet to be re-treated. One patient with genotype 1 infection treated with SIM + SOF was negative for NS5B resistance mutations and is planned to initiate SOF + velpatasvir with RBV. Three patients with genotype 1 HCV treated with 12 weeks of SIM + SOF did not undergo viral resistance mutation testing but were successfully re-treated with LDV + SOF ± RBV with SVR. One patient initially treated with 16 weeks of SOF and RBV for genotype 2 infection was subsequently treated with 12 weeks of SOF + velpatasvir with SVR. Finally, one patient with genotype 1 infection treated with LDV + SOF was lost to follow-up and did not undergo resistance testing or re-treatment.

SVR was observed in 93% of patients without cirrhosis, 88% of patients with compensated cirrhosis, and 82% of patients with decompensated cirrhosis. Female sex was associated with higher rates of SVR. Rates of SVR were numerically but not significantly higher in racial/ethnic minority patients compared to Whites (91% vs. 87%, *P* = 0.15), HIV-negative patients compared to HIV-infected patients (90% vs. 87%, *P* = 0.51), patients without drug or alcohol history compared to those with drug or alcohol abuse (92% vs. 89%, *P* = 0.34), and those without a history of mental health disorders compared to those with mental health disorders (90% vs. 89%, *P* = 0.57). There was no difference in rates of SVR in insured and uninsured patients (90% vs. 90%, *P* = 0.98). After adjustment, decompensated cirrhosis was the only significant negative predictor of SVR (Table 2).

**Table 2:** Predictors of SVR12 by univariate and multivariate logistic regression analysis

Variable	Univariate analysis (OR, 95% CI)	Multivariate analysis (OR, 95% CI)	Absolute SVR12 rates (%)
Female sex (N = 224)	1.73 (0.95–3.19)	1.64 (0.89–3.02)	92% (vs. male sex 88%)
Cirrhosis status			
No cirrhosis (N = 250)	Reference	Reference	92.8%
Compensated cirrhosis (N = 206)	0.56 (0.30–1.06)	0.60 (0.32–1.14)	87.9%
Decompensated cirrhosis (N = 56)	0.36 (0.15–0.82)	0.37 (0.16–0.85)	82.1%

Statistical significance defined as *P* < 0.10 for univariate and *P* < 0.05 for multivariate analysis

*SVR12* sustained virologic response at 12 weeks after treatment end, *OR* odds ratio, *CI* confidence interval

## Discussion

In this retrospective cohort study, we evaluated the effectiveness of DAA-based treatment for HCV in a predominantly underinsured, racial/ethnic minority population, with high rates of alcohol and substance use, mental health disorders, and high rates of cirrhosis – a group of patients who must be treated in order to achieve HCV eradication. We found that 90% of this traditionally “difficult-to-treat” population followed in a resource-constrained safety-net health system could achieve SVR with DAA-based therapy. There were no differences in SVR by insurance status, racial/ethnic group, or history of drug/alcohol use. The only factor negatively associated with SVR was the presence of decompensated cirrhosis, highlighting another benefit of treating patients with earlier stages of fibrosis prior to disease progression [21].

We found a high rate of treatment success in our cohort comparable to those reported in other real-world outcome studies at large academic centers and private clinics [9–12]. Our population is unique in that it consists of a large group of low socioeconomic status patients, of whom 56% were uninsured and 13% were Medicaid insured. This demographic has a considerably higher prevalence of chronic HCV infection than the general population [22], yet is consistently under-represented in existing real-world studies of DAA treatment outcomes. Only two smaller studies have described effectiveness of DAAs in indigent populations [14, 23]. Barocas et al. [23] reported SVR was observed in 62 (97%) of 64 homeless and marginally housed adults; however, included patients had been pre-selected based on likelihood of adherence to treatment and all but one were insured. Beck et al. [14] reported SVR in 183 (93%) of 189 patients receiving SOF-based HCV therapy within a safety-net health system in San Francisco; however, 95% of patients in this study were also insured. Of note, both prior studies were conducted in states that offer expanded Medicaid, through which access to HCV treatment is possible for a larger proportion of low-income patients. Data shows that the burden of HCV is 7.5% higher in those with Medicaid compared to commercial insurance [24]. In states without expanded Medicaid, such as Texas, access to HCV treatment is limited due to a smaller proportion of low-income patients being granted Medicaid coverage, as well as the existence of restrictions on treatment eligibility (i.e., advanced liver disease, negative toxicology screens, etc.) for those who do have Medicaid coverage. Our study data represent outcomes that can be achieved even in a state without expanded Medicaid. Further, we found that rates of SVR were unaffected by insurance status, suggesting that concerns regarding treatment of underinsured populations may be unfounded.

The success of our program is based on (1) utilizing resources available through pharmaceutical companies, namely through PAPs, which are designed to provide access to medications for those without insurance; and (2) coordinating comprehensive care through a multi-disciplinary team consisting of pharmacists, nurse navigators, and providers. In our set up, pharmacists or pharmacy technicians assisted patients in completing medication application forms, collating necessary documentation, obtaining insurance prior authorization, and arranging medication delivery to patient homes. Our nurse navigator coordinated follow-up blood draws and clinic visits. Several improvements were made to the process as the clinic expanded, wherein visits arranged after treatment to assess for SVR (“SVR visits”) were found to reduce loss to follow-up; linking of laboratory and appointment visits such that they occurred on the same day improved patient experience; education of primary care providers within the healthcare system on HCV therapies and the clinic referral process improved referral rates; and introduction of an order set that combined medication and laboratory orders allowed monthly medication dispensing and follow-up blood draws to occur in a timely fashion, thereby reducing gaps in treatment. Through strategic planning and resource utilization, our clinic effectively adapted minimal resources to screen and initiate DAAs at a rate of approximately 50 new patients per month. This model may be particularly helpful in centers caring for large numbers of uninsured patients, amongst whom the prevalence of chronic HCV infection is considerably higher than in the general population [22].

Another unique feature of our patient population is the high proportion of Black and Hispanic patients (44% and 16%, respectively) as compared to other real-world studies, reflecting the ethnic makeup of the patient population within the Parkland catchment area. African Americans and Hispanics in the USA are disproportionately affected by HCV [13]. Previous studies found that disease progression tends to be accelerated in Hispanic patients [25], and both African Americans and Hispanics are more likely to experience treatment failure with IFN-based therapy as compared to their White counterparts [26, 27]. A recent study of 21,095 Veterans treated with DAAs found Blacks and Hispanics were less likely to respond to DAAs compared to Whites after adjusting for baseline characteristics, especially if using shorter 8-week courses of treatment [28]. However, in our population, we found that treatment with DAA-based therapy yielded similar rates of success in Black, Hispanic, and White patient groups.

We found decompensated cirrhosis to be the only negative predictor of SVR in multivariate analysis, which has similarly been described in prior analyses [9–11]. Our uninsured patients were treated regardless of stage of liver disease because predictive models have demonstrated the cost efficiency of treating individuals with earlier stage disease [21] and that curative treatment improves quality of life and mortality [18]. However, Medicaid reimbursement criteria in Texas (and many other states) restrict DAA accessibility to patients with advanced liver disease. Additional Medicaid reimbursement criteria in other states include evidence of undetectable HIV RNA in co-infected patients and documented drug and alcohol abstinence [29]. Our study found similar rates of treatment success among patients with alcohol and drug use histories as well as those co-infected with HIV, although data quantifying active substance use and HIV RNA levels in co-infected patients was not collected. These data suggest that imposing restrictions on access to DAAs based on stage of liver disease, HIV status, and substance use is not evidence-based and contributes to socioeconomic disparity, disrupts prevention efforts, and ultimately leads to increased healthcare expenditure [29, 30]. Patients denied HCV treatment by Medicaid are unlikely to qualify for PAPs because they are considered to be otherwise “funded”, thereby creating a paradoxical situation in which some patients on Medicaid are disadvantaged in terms of HCV treatment access compared to those without insurance.

Our study population suffered a loss to follow-up rate of 5%, only marginally higher than the 2.5–3.8% reported in non-indigent real-world studies [9–11] and comparable with the 5–15% reported in previous studies of IFN-based therapy in indigent populations [24, 28, 31]. Interestingly, among those lost to liver clinic follow-up, approximately 1 in 5 were subsequently seen by their primary care providers within the same healthcare system. It is possible that knowledge gaps in non-specialist providers were a contributing factor to the lack of laboratory follow-up after treatment completion; thus, future development of targeted interventions, such as provider education on HCV screening and treatment, may help to overcome these barriers.

This study had several key limitations. Firstly, it was an observational, non-randomized retrospective study without a control group, and was underpowered to fully examine the relative benefits and drawbacks of different DAA regimens. Second, our clinic structure included specialist providers, and thus our findings may not translate to other primary care practice settings. Finally, long-term follow-up data with which to identify reinfection rates was unavailable; such data may play an important role in this high-risk population.

## Conclusion

The advent of DAAs has led to improved outcomes amongst those with access to treatment. However, underinsured patients who are cared for by safety-net hospitals carry a significant burden of disease but may have limited access to these curative therapies. Our program demonstrates that access to HCV treatment is possible with a dedicated staff, and that outcomes in this socioeconomically disadvantaged population are comparable to those achieved in commercially insured, high-income settings.

## Additional file

Additional file 1: Table S1. Treatment regimen by HCV genotype. Table of direct-acting antiviral regimen administered for each HCV genotype subgroup. (XLSX 58 kb)
